# Supplementary material for: Patterns of Intron Gain and Loss in Fungi
Source: PLoS Biol. 2004 Nov 30;2(12):e422. doi: 10.1371/journal.pbio.0020422 (PMC532390; doi:10.1371/journal.pbio.0020422)
Supplement: Table S1 — Also available at http://genes.mit.edu/NielsenEtAl/. (4.3 MB ZIP). [file pbio.0020422.st001.zip › NielsenEtAl/html/953.html]

AN7649.1.NCU00198.1.MG06427.1.FG05543.1


```
 CLUSTAL W (1.82) Multiple Sequence Alignments - Introns Inserted


Sequence 1: NCU00198.1	88 aa
Sequence 2: FG05543.1	87 aa
Sequence 3: MG06427.1	87 aa
Sequence 4: AN7649.1	90 aa
Alignment Length: 90 aa
Number Identitical Residues: 32 aa
Alignment Score (without introns) 1920


MG06427.1 	MDQ2LNNNEMRELEQRMQKRQVKEFVN0IFSKLVDHCFMSCIDDFSSKALNSRESGCIQR
NCU00198.1	MDG2LTAAESRELDQRLQKRQVKEFMS0VFGNLVDNCFTACVDDFTSKALSGRESGCISR
FG05543.1 	MDM2LSAAEQRTLEQRMQKRQVKEFMG0AFGGLVEHCFMSCVDDFTSKAISNRESGCINR
AN7649.1  	MPR~LNAAEQRELANRMERKQMKEFMT0MYSKLVQRCFDDCVNDFTTKSLISREEGCVMR
          	*   *.  * * * :*::::*:***:   :. **:.**  *::**::*:: .**.**: *

MG06427.1 	CVIKDMAAQQRLGERFQELSAQMANQQQQ---
NCU00198.1	CVLKSMSTQTRLGERFGELNAAMTAEMQRR--
FG05543.1 	CVQKWMASQQRISDRFQEHNAQLTAQMNK---
AN7649.1  	CVDKFMKGSQRLNERFQEQNAAMMQSGQLPGR
          	** * *  . *:.:** * .* :  . :  .
```
